# List 3-coloring on comb-convex and caterpillar-convex bipartite graphs

**DOI:** 10.1007/s10878-026-01424-5

**Published:** 2026-05-16

**Authors:** Banu Baklan Şen, Thomas Erlebach, Öznur Yaşar

**Affiliations:** 1 Computer Engineering Department, Istanbul Nisantasi University, Istanbul, Turkey; 2 Department of Computer Science, Durham University, Durham, United Kingdom; 3 Computer Engineering Department, Kadir Has University, Istanbul, Turkey

**Keywords:** Caterpillar-convex bipartite graphs, Comb-convex bipartite graphs, Computational complexity, List coloring, 68R10, 68Q25

## Abstract

Given a graph $G=(V, E)$ and a list of available colors *L*(*v*) for each vertex $v\in V$, where $L(v) \subseteq \{1, 2, \ldots , k\}$, List *k*-Coloring refers to the problem of assigning colors to the vertices of *G* such that each vertex receives a color from its own list and no two neighboring vertices receive the same color. The decision version of the problem List 3-Coloring is NP-complete even for bipartite graphs, and its complexity on comb-convex bipartite graphs has been an open problem. We give a polynomial-time algorithm to solve List 3-Coloring for caterpillar-convex bipartite graphs, a superclass of comb-convex bipartite graphs. We also give a polynomial-time recognition algorithm for the class of caterpillar-convex bipartite graphs.

## Introduction

Graph coloring is the problem of assigning colors to the vertices of a given graph in such a way that no two adjacent vertices have the same color. *List coloring* (Vizing 1976; Erdős et al. 1979) is a generalization of graph coloring in which each vertex must receive a color from its own list of allowed colors. In this paper, we study the list coloring problem with a fixed number of colors in subclasses of bipartite graphs. We give a polynomial-time algorithm for the list 3-coloring problem for caterpillar-convex bipartite graphs, a superclass of comb-convex bipartite graphs. We also give a polynomial-time recognition algorithm for the class of caterpillar-convex bipartite graphs. Our results resolve the open question regarding the complexity of list 3-coloring for comb-convex bipartite graphs stated by Bonomo-Braberman et al. (2021, 2024).

We consider finite simple undirected graphs $G=(V, E)$ with vertex set *V* and edge set *E*. By $N_G(v)$ (or by *N*(*v*) if the graph is clear from the context) we denote the neighborhood of *v* in *G*, i.e., the set of vertices that are adjacent to *v*. A *k-coloring* of *G* is a labeling that assigns colors from the set $[k]=\{1, 2, \ldots , k\}$ to the vertices of *G*. A coloring is *proper* if no two adjacent vertices have the same color. A *list assignment* of a graph $G=(V, E)$ is a mapping $\mathcal {L}$ that assigns each vertex $v \in V$ a list $\mathcal {L}(v) \subseteq \{1, 2,\ldots \}$ of admissible colors for *v*. When $\mathcal {L}(v) \subseteq [k]=\{1, 2,\ldots k\}$ for every $v \in V$ we say that $\mathcal {L}$ is a *k*-list assignment of *G*. The total number of available colors is bounded by *k* in a *k*-list assignment. On the other hand, when the only restriction is that $|\mathcal {L}(v)| \le k$ for every $v \in V$, then we say that $\mathcal {L}$ is a list *k*-assignment of *G*. *List coloring* is the problem of deciding, for a given graph $G=(V,E)$ and list assignment $\mathcal {L}$, whether *G* has a proper coloring where each vertex *v* receives a color from its list $\mathcal {L}(v)$. If $\mathcal {L}$ is a *k*-list assignment for a fixed value of *k*, the problem becomes the list *k*-coloring problem:

$$\underline{{\textsc {List}}\,k-{\textsc {Coloring}}\,({\textsc {Li}}\,k-{\textsc {Col}})}$$

*Instance:* A graph $G=(V,E)$ and a *k*-list assignment $\mathcal {L}$.

*Question:* Does *G* have a proper coloring where each vertex *v* receives a color from its list $\mathcal {L}(v)$?

If $\mathcal {L}$ is a list *k*-assignment instead of a *k*-list assignment, the problem is called *k*-List Coloring.

**Fig. 1:**
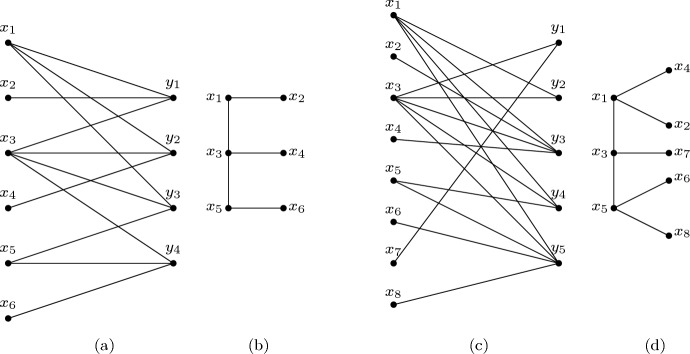
(a) A comb-convex bipartite graph $G_1$, (b) a comb representation of $G_1$, (c) a caterpillar-convex bipartite graph $G_2$, and (d) a caterpillar representation of $G_2$

The classes of bipartite graphs of interest to us are defined via a convexity condition for the neighborhoods of the vertices on one side of the graph with respect to a tree defined on the vertices of the other side. The following types of trees are relevant here: A *star* is a tree of diameter at most 2. A *comb* is a tree that consists of a chordless path *P*, called the *backbone*, with a single leaf neighbor attached to each backbone vertex (Chen et al. 2016). A *caterpillar* is a tree that consists of a chordless path *P*, called the *backbone*, with an arbitrary number (possibly zero) of leaf vertices attached to each vertex on *P*. Note that if a caterpillar has exactly one leaf vertex attached to each vertex on *P*, then that caterpillar is a comb.

A bipartite graph $G=(X\cup Y,E)$ is called a *star-convex* (or *comb-convex*, or *caterpillar-convex*) bipartite graph if a star (or comb, or caterpillar) $T=(X,F)$ can be defined on *X* such that for each vertex $y \in Y$, its neighborhood $N_G(y)$ induces a subtree of *T*. The star (or comb, or caterpillar) $T=(X,F)$ is then called a *star representation* (or *comb representation*, or *caterpillar representation*) of *G*.

Figure 1 shows an example of a comb-convex bipartite graph and its comb representation, and a caterpillar-convex bipartite graph and its caterpillar representation. Both the comb (in part (b)) and the caterpillar (in part (d)) have the path $P=x_1 x_3 x_5$ as backbone.

The remainder of this paper is organized as follows. In Section 2, we discuss related work. In Section 3, we give a polynomial-time algorithm for Li $3$-col for caterpillar-convex bipartite graphs (and thus also for comb-convex bipartite graphs). In Section 4, we give a polynomial-time recognition algorithm for caterpillar-convex bipartite graphs. In Section 5, we give concluding remarks.

## Related work

Deciding whether a graph has a proper coloring with *k* colors is polynomial-time solvable when $k=1$ or 2 (Lovász 1973) and NP-complete for $k\ge 3$ (Vizing 1976). As Li $k$-col generalizes this problem, it is also NP-complete for $k\ge 3$. When the list coloring problem is restricted to perfect graphs and their subclasses, it is still NP-complete in many cases such as for bipartite graphs (Kubale 1992) and interval graphs (Biró et al. 1992). On the other hand, it is polynomially solvable for trees and graphs of bounded treewidth (Jansen and Scheffler 1997). The problems Li *k*-col and *k*-List Coloring are polynomial-time solvable if $k\le 2$ and NP-complete if $k\ge 3$ (Lovász 1973; Vizing 1976). *k*–list coloring has been shown to be NP-complete for small values of *k* for complete bipartite graphs and cographs by Jansen and Scheffler (1997), as observed by Golovach and Paulusma (2014). The 3-List Coloring problem is NP-complete even if each color occurs in the lists of at most three vertices in planar graphs with maximum degree three, as shown by Kratochvíl and Tuza (1994).

We use the following standard notation for specific graphs: $P_t$ denotes a path with *t* vertices; $K_t$ denotes a clique with *t* vertices; $K_{\ell ,r}$ denotes a complete bipartite subgraph with parts of sizes $\ell$ and *r*; $K_{1,s}^1$ denotes the 1-subdivision of $K_{1,s}$ (i.e., every edge $e=\{u,v\}$ of $K_{1,s}$ is replaced by two edges $\{u,w_e\}$ and $\{w_e,v\}$, where $w_e$ is a new vertex); and $sP_1 + P_5$ is the disjoint union of *s* isolated vertices and a $P_5$. Li $k$-col is known to be NP-complete even for $k = 3$ within the class of 3–regular planar bipartite graphs (Kratochvil 1993). On the positive side, for fixed $k \ge 3$, Li *k*-col is polynomially solvable for $P_5$-free graphs (Hoàng et al. 2010). Li 3-col is polynomial-time solvable for $P_6$-free graphs (Broersma et al. 2013) and for $P_7$-free graphs (Bonomo et al. 2018). Li 3-col is polynomial-time solvable for $(K^1_{1,s}, P_t)$-free graphs for every $s \ge 1$ and $t \ge 1$ (Chudnovsky et al. 2020). Li *k*-col is polynomial-time solvable for $(sP_1 + P_5)$-free graphs, which was proven for $s = 0$ by Hoàng et al. (2010) and for every $s \ge 1$ by Couturier et al. (2015).

**Fig. 2:**
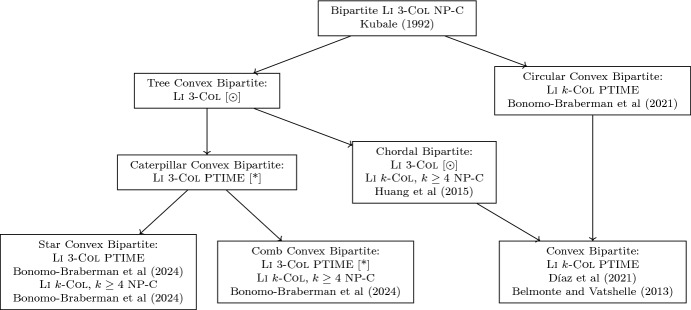
Computational complexity results for Li *k*-Col on subclasses of bipartite graphs. [*] refers to this paper, [$\odot$] refers to an open problem, NP-C denotes NP-complete problem, PTIME denotes polynomial-time solvable problem

An overview of complexity results for Li $k$-col in some subclasses of bipartite graphs is shown in Fig. 2. The computational complexity of Li 3-col for chordal bipartite graphs has been stated as an open problem in 2015 (Huang et al. 2015) and has been of interest since then (Díaz et al. 2021). Díaz et al. (2021) give a partial answer to this question by showing that Li *k*-col is polynomial-time solvable in the class of biconvex bipartite graphs and convex bipartite graphs. Li *k*-col is solvable in polynomial time when it is restricted to graphs with all connected induced subgraphs having a multichain ordering (Enright et al. 2014). This result can be applied to permutation graphs and interval graphs. Díaz et al. (2021) show that connected biconvex bipartite graphs have a multichain ordering, implying a polynomial-time algorithm for Li *k*-col on this graph class. They also provide a dynamic programming algorithm to solve Li *k*-col in the class of convex bipartite graphs and show how to modify the algorithm to solve the more general Li *H*-col problem on convex bipartite graphs. As observed by Bonomo-Braberman et al. (2024), the fact that Li *k*-col can be solved in polynomial time on convex bipartite graphs also follows because the mim-width of convex bipartite graphs is bounded and quickly computable as shown by Belmonte and Vatshelle (2013). The computational complexity of Li 3-col for $P_8$-free bipartite graphs is open (Biró et al. 1992). Even the restricted case of Li 3-col for $P_8$-free chordal bipartite graphs is open. Golovach and Paulusma (2014) survey results for Li $k$-col on *H*-free graphs in terms of the structure of *H*.

So-called *width parameters* play a crucial role in algorithmic complexity. For various combinatorial problems, it is possible to find a polynomial-time solution by exploiting bounded width parameters such as mim-width, sim-width and clique-width. Given a graph class, it is known that when mim-width is bounded and quickly computable, then Li *k*-col is polynomial-time solvable (Brettell et al. 2022). Brettell et al. (2022) proved that for every $r \ge 1, s \ge 1$ and $t \ge 1$, the mim-width is bounded and quickly computable for $(K_r, K^1_{1,s}, P_t)$-free graphs. This result further implies that for every $k \ge 1, s \ge 1$ and $t \ge 1$, Li *k*-col is polynomial-time solvable for $(K^1_{1,s}, P_t)$-free graphs. Most recently, Bonomo-Braberman et al. (2021) showed that mim-width is unbounded for star-convex and comb-convex bipartite graphs. On the other hand, Li 3-col is polynomial-time solvable for star-convex bipartite graphs whereas Li *k*-col is NP-complete for $k\ge 4$ (Bonomo-Braberman et al. 2024). Furthermore, Bonomo-Braberman et al. (2024) show that for comb-convex bipartite graphs, Li *k*-col remains NP-complete for $k\ge 4$ and leave open the computational complexity of Li 3-col for this graph class. In this paper, we resolve this problem by showing that Li 3-col is polynomial-time solvable even for caterpillar-convex bipartite graphs. Bonomo-Braberman et al. (2024) also consider $(t, \varDelta )$-tree convex bipartite graphs for $t,\varDelta \in \mathbb {N} \cup \{\infty \}$ with $t \ge 1$, $\varDelta \ge 3$, where a tree is a $(t,\varDelta )$-tree if its maximum degree is bounded by $\varDelta$ and it contains at most *t* vertices of degree at least 3. They show that the class of $(t,\varDelta )$-tree convex bipartite graphs has bounded mim-width if and only if $\{\varDelta ,t\}\cap \{\infty \}=\emptyset$, Furthermore, they show that Li *k*-col for $k\ge 4$ for $(t,\varDelta )$-tree convex bipartite graphs is polynomial-time solvable if $\{\varDelta ,t\}\cap \{\infty \}=\emptyset$ and NP-complete otherwise. To the best of our knowledge, the complexity or Li 3-col for tree-convex bipartite graphs remains open.

As for the recognition of graph classes, Bonomo-Braberman et al. (2024) provide an algorithm for the recognition of $(t, \varDelta )$-tree convex bipartite graphs by using a result by Buchin et al. (2011). This result for the recognition of $(t, \varDelta )$-tree convex bipartite graphs, however, does not apply to caterpillar-convex bipartite graphs. Therefore, we give a novel algorithm for the recognition of caterpillar-convex bipartite graphs.

## List 3-coloring caterpillar-convex bipartite graphs

In this section we give a polynomial-time algorithm for solving Li $3$-col in caterpillar-convex bipartite graphs. Let a caterpillar-convex bipartite graph $G=(X\cup Y,E)$ be given, together with a 3-list assignment $\mathcal {L}$. We assume that a caterpillar $T=(X,F)$ is also given, where $N_G(y)$ induces a subtree of *T* for each $y\in Y$. If the caterpillar is not provided as part of the input, we can compute one in polynomial time using the recognition algorithm that we present in Section 4.

Let *T* consist of a backbone *B* with vertices $b_1,b_2,\ldots ,b_n$ (in that order) and a set of leaves $L(b_i)$, possibly empty, attached to each $b_i\in B$. We use *L* to denote the set of all leaves, i.e., $L=\bigcup _{i=1}^n L(b_i)$. Furthermore, for any $1\le i\le j \le n$, we let $B_{i,j}=\{b_i,b_{i+1},\ldots ,b_j\}$ and $L_{i,j}=\bigcup _{k=i}^j L(b_k)$.

The idea of the algorithm is to define suitable subproblems that can be solved in polynomial time, and to obtain the overall coloring as a combination of solutions to subproblems. Roughly speaking, the subproblems consider stretches of the backbone in which all backbone vertices are assumed to be assigned the same color in a proper list 3-coloring. More precisely, a subproblem $$SP(i,j,c_1,c_2,c_3)$$ is specified via two values *i*, *j* with $$1\le i\le j\le n$$ and three colors $$c_1,c_2,c_3$$ with $$c_1\ne c_2$$ and $$c_2\ne c_3$$ where $$c_i \in [3],$$ for $$ i=1, 2, 3$$. Hence, there are $$O(n^2)$$ subproblems.

**Fig. 3:**
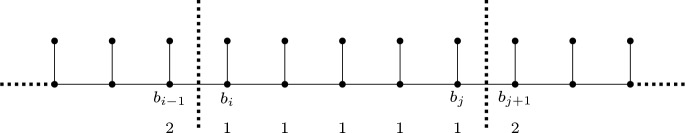
Illustration of subproblem $SP(i,j,c_1,c_2,c_3)$ for the case *SP*(*i*, *j*, 2, 1, 2)

The subproblem $S=SP(i,j,c_1,c_2,c_3)$ is concerned with the subgraph $G_S$ of *G* induced by $B_{i-1,j+1}\cup L_{i,j} \cup \{y\in Y\mid N(y)\cap (B_{i,j}\cup L_{i,j})\ne \emptyset \}$. It assumes that color $c_1$ is assigned to $b_{i-1}$, color $c_2$ to the backbone vertices from $b_i$ to $b_j$, and color $c_3$ to $b_{j+1}$. See Fig. 3 for an illustration of *SP*(*i*, *j*, 2, 1, 2). Solving the subproblem *S* means determining whether this coloring of $B_{i-1,j+1}$ can be extended to a proper list 3-coloring of $G_S$. The result of the subproblem is False if this is not possible, or True (along with a suitable proper list 3-coloring of $G_S$) otherwise. If $c_1\notin \mathcal {L}(b_{i-1})$, or $c_3\notin \mathcal {L}(b_{j+1})$, or $c_2\notin \mathcal {L}(b_k)$ for some $i\le k\le j$, then the result of the subproblem is trivially False.

We will show that this subproblem can be solved in polynomial time as it can be reduced to the 2-list coloring problem, which is known to be solvable in linear time (Edwards 1986; Gravier et al. 2002). Furthermore, solutions to consecutive ‘compatible’ subproblems can be combined, and a proper list 3-coloring of *G* exists if and only if there is a collection of subproblems whose solutions can be combined into a list 3-coloring of *G*. For example, the colorings of two subproblems $SP(i,j,c_1,c_2,c_3)$ and $SP(j+1,k,c_2,c_3,c_4)$ can be combined because they agree on the colors of backbone vertices that are in both subproblems, they do not share any leaf vertices, and the vertices $y\in Y$ that have neighbors in both $B_{i,j}\cup L_{i,j}$ and $B_{j+1,k}\cup L_{j+1,k}$ must be adjacent to $b_{j}$ and $b_{j+1}$, which are colored with colors $c_2$ and $c_3$ (where $c_2\ne c_3$) in the colorings of both subproblems, and hence must have received the same color (the only color in $\{1,2,3\}\setminus \{c_2,c_3\}$) in both colorings. To check whether there is a collection of compatible subproblems whose solutions can be combined into a list 3-coloring of *G*, we will show that it suffices to search for a directed path between two vertices in an auxiliary directed acyclic graph (DAG) on the subproblems whose result is True.

For a subproblem $S=SP(i,j,c_1,c_2,c_3)$, if $i=1$, there is no vertex $b_{i-1}$, and we write $*$ for $c_1$; similarly, if $j=n$, there is no vertex $b_{j+1}$, and we write $*$ for $c_3$. The graph $G_S$ considered when solving such a subproblem does not contain $b_{i-1}$ or $b_{j+1}$, respectively, but is otherwise defined analogously. If $i=1$ and $j=n$, then $G_S$ contains neither $b_{i-1}$ nor $b_{j+1}$.

### Lemma 1

There is a linear-time algorithm for solving any subproblem of the form $SP(i,j,c_1,c_2,c_3)$.

### Proof

Consider the subproblem $S=SP(i,j,c_1,c_2,c_3)$. Let $G_S$ be the subgraph of *G* defined by *S*, and let $X_S\subseteq X$, $Y_S\subseteq Y$ be such that the vertex set of $G_S$ is $X_S\cup Y_S$. First, we check whether $c_1\in \mathcal {L}(b_{i-1})$ (only if $i>1$), $c_3\in \mathcal {L}(b_{j+1})$ (only if $j<n$), and $c_2\in \mathcal {L}(b_k)$ for all $i\le k\le j$. If one of these checks fails, we return False. Otherwise, we assign color $c_1$ to $b_{i-1}$, color $c_2$ to all vertices in $B_{i,j}$, and color $c_3$ to $b_{j+1}$.

For every vertex $y\in Y_S$, we check if *N*(*y*) contains any vertices of $B_{i-1,j+1}$ and, if so, remove the colors of those vertices from $\mathcal {L}(y)$ (if they were contained in $\mathcal {L}(y)$). If the list of any vertex $y\in Y_S$ becomes empty in this process, we return False.

Let $B_S$ consist of the backbone vertices in $X_S$ and $L_S$ consist of the leaf vertices in $X_S$ (with respect to the caterpillar *T*). If there is a vertex in $L_S$ or $Y_S$ with a list of size 1, assign the color in that list to that vertex and remove that color from the lists of its neighbors (if it is contained in their lists). Repeat this operation until there is no uncolored vertex with a list of size 1. (If an uncolored vertex with a list of size 1 is created later on in the algorithm, the same operation is applied to that vertex.) If the list of any vertex becomes empty in this process, return False. Otherwise, we must arrive at a state where all uncolored vertices in $G_S$ have lists of size 2 or 3.

If there is a vertex $y\in Y_S$ with a list of size 3, that vertex must be adjacent to a single leaf $\ell$ in $L_S$ (as it cannot be adjacent to a backbone vertex). In this case we remove an arbitrary color from $\mathcal {L}(y)$: This is admissible as, no matter what color $\ell$ receives in a coloring, vertex *y* can always be colored with one of the two colors that have remained in its list.

If there is a vertex $\ell \in L_S$ with a list of length 3, assign color $c_2$ to $\ell$ (and remove color $c_2$ from the lists of vertices in $N(\ell )$). This color assignment does not affect the existence of a proper list 3-coloring for the following reasons (where we let $b_k$ denote the backbone vertex with $\ell \in L(b_k)$):

- If a vertex $y\in N(\ell )$ is adjacent to more than one vertex, it must be adjacent to $b_k$, which has been colored with $c_2$, and hence it cannot receive color $c_2$ in any case.
- If a vertex $y\in N(\ell )$ is adjacent only to $\ell$ and no other vertex, then *y* can still be colored after $\ell$ is assigned color $c_2$, because we cannot have $\mathcal {L}(y)=\{c_2\}$; this is because, if *y* had the list $\mathcal {L}(y)=\{c_2\}$, it would have been colored $c_2$ and the color $c_2$ would have been removed from $\mathcal {L}(\ell )$.

If at any step of this process, an uncolored vertex with an empty list is created, return False. Otherwise, we arrive at an instance *I* of Li $3$-col where all uncolored vertices have lists of size 2. Such an instance can be solved in linear time (Edwards 1986; Gravier et al. 2002) (via reduction to a 2SAT problem). If *I* admits a proper list 3-coloring, that coloring gives a proper list 3-coloring of $G_S$, and we return True and that coloring. Otherwise, we return False.

Correctness of the algorithm follows from its description, and the algorithm can be implemented to run in linear time using standard techniques. $\square$

**Algorithm 1:**
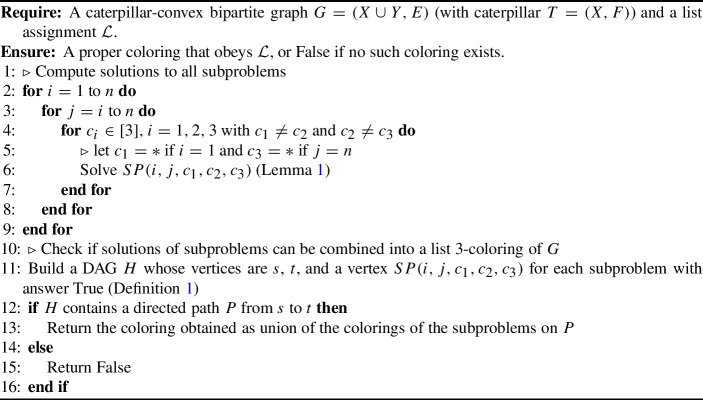
List-3-Coloring Algorithm for Caterpillar-Convex Bipartite Graphs

Call a subproblem $S=SP(i,j,c_1,c_2,c_3)$ *valid* if its answer is True (and a proper list 3-coloring of $G_S$ has been produced), and *invalid* otherwise. To check whether the colorings obtained from valid subproblems can be combined into a list 3-coloring of *G*, we make use of an auxiliary DAG *H* constructed as follows. The existence of a proper list 3-coloring of *G* can then be determined by checking whether *H* contains a directed path from *s* to *t*.

### Definition 1

The auxiliary DAG $H=(V_H,A)$ has vertices *s*, *t*, and a vertex for each valid subproblem $SP(i,j,c_1,c_2,c_3)$. Its arc set *A* contains the following arcs: An arc $(s,SP(1,i,*,c_2,c_3))$ for each $i<n$ and $c_2,c_3\in [3]$ such that $SP(1,i,*,c_2,c_3)$ is valid; an arc $(SP(i,n,c_1,c_2,*),t)$ for each $i>1$ and $c_1,c_2\in [3]$ such that $SP(i,n,c_1,c_2,*)$ is valid; arcs $(s,SP(1,n,*,c_2,*))$ and $(SP(1,n,*,c_2,*),t)$ if $SP(1,n,*,c_2,*)$ is valid; an arc $(SP(i,j,c_1,c_2,c_3),SP(j+1,k,c_2,c_3,c_4)$ for each $i\le j\le k-1$ and each $c_1,c_2,c_3,c_4\in [3]$ (or $c_1=*$ or $c_4=*$ if $i=1$ or $k=n$, respectively) such that $SP(i,j,c_1,c_2,c_3)$ and $SP(j+1,k,c_2,c_3,c_4)$ are both valid.

### Theorem 1

Li $3$-col can be solved in polynomial time for caterpillar-convex bipartite graphs.

### Proof

Let a caterpillar-convex bipartite graph $G=(X\cup Y,E)$ with caterpillar representation $T=(X,F)$ be given. Let *n* denote the number of backbone vertices in *T*, and $|G|=|X|+|Y|+|E|$ the size of *G*. The algorithm, shown in Algorithm 1, first computes the solutions to all $O(n^2)$ subproblems $SP(i,j,c_1,c_2,c_3)$. This can be done in linear time *O*(|*G*|) per subproblem (Lemma 1), and thus in time $O(n^2 |G|)$ overall.

Then it constructs the auxiliary DAG *H* (Definition 1) and checks if *H* contains a path *P* from *s* to *t*. As *H* contains $O(n^2)$ vertices and $O(n^3)$ edges, the construction of *H* and the check for the existence of an *s*-*t* path can be carried out in $O(n^3)$ time.

Finally, if an *s*-*t* path *P* is found, the colorings corresponding to the subproblems on *P* can be combined into a list 3-coloring of *G* in *O*(|*G*|) time. Thus, the overall running-time of the algorithm can be bounded by $O(n^2|G|)$, which is polynomial in the size of the input. If the caterpillar representation $T=(X,F)$ is not given as part of the input, it can be computed via our recognition algorithm (Section 4) in polynomial time (the proof of Theorem 2 shows that the time for computing $T=(X,F)$ is at most $O(|X\cup Y|^3)$).

To show that the algorithm is correct, assume first that the algorithm finds an *s*-*t* path *P* in *H*. Let $\mathcal {S}$ be the set of valid subproblems on *P*. By construction, each backbone vertex receives the same color in the at most three subproblems in $\mathcal {S}$ in which it occurs. Each leaf of the caterpillar occurs in exactly one subproblem in $\mathcal {S}$. Every vertex in *Y* that occurs in more than one subproblem in $\mathcal {S}$ must receive the same color in each such subproblem (because it must be adjacent to the two backbone vertices with different colors at the border between any two consecutive subproblems in which it is contained). The other vertices in *Y* occur in only one subproblem. Hence, the coloring obtained by the algorithm is a proper list 3-coloring of *G*.

For the other direction, assume that *G* admits a proper list 3-coloring. Then partition the backbone $b_1,b_2,\ldots ,b_n$ into maximal segments $B_{1,i_1}, B_{i_1+1,i_2}, \ldots ,$ $B_{i_{k-1}+1,i_k}$ for some $k\ge 1$ and $i_k=n$, so that all backbone vertices in each segment receive the same color. Let the color of the backbone vertices in $B_{i_{j-1}+1,i_j}$ be $c_j$ (where $i_{j-1}=0$ if $j=1$), for $1\le j\le k$. This implies that the subproblems $SP(1,i_1,*,c_1,c_2)$, $SP(i_1+1,i_2,c_1,c_2,c_3)$, ..., $SP(i_{k-1}+1,i_k,c_{k-1},c_k,*)$ are all valid and constitute an *s*-*t*-path in the DAG *H*. Therefore, the algorithm will output a proper list 3-coloring. $\square$

As comb-convex bipartite graphs are a subclass of caterpillar-convex bipartite graphs, we obtain:

### Corollary 1

Li 3-col can be solved in polynomial time for comb-convex bipartite graphs.

Combining Corollary 1 with Theorem 4 from Bonomo-Braberman et al. (2024) and the polynomial-time solvability of Li *k*-col for $k\le 2$ (Erdős et al. 1979; Vizing 1976) yields a complexity dichotomy: Li *k*-col is polynomial-time solvable on comb-convex bipartite graphs when $k\le 3$; otherwise, it is NP-complete.

## Recognition of caterpillar-convex bipartite graphs

We give a polynomial-time recognition algorithm for caterpillar-convex bipartite graphs. We are given a bipartite graph $G=(X\cup Y,E)$ and want to decide whether it is caterpillar-convex and, if so, construct a caterpillar representation $T=(X,F)$. First, we assume that a specific partition of the vertex set into independent sets *X* and *Y* is given as part of the input, and we want to decide whether there is a caterpillar representation $T=(X,F)$ with respect to that given bipartition (i.e., the vertex set of the caterpillar is the independent set *X* that was specified in the input). At the end of this section, we will discuss how to handle the case that the bipartite graph is given without a specific bipartition of the vertex set and we want to decide whether the vertex set can be partitioned into independent sets *X* and *Y* in such a way that there is a caterpillar representation with respect to that bipartition.

In a DAG, a vertex with no outgoing arcs is called a *sink*. The main idea of the algorithm for recognizing caterpillar-convex bipartite graphs is to construct an auxiliary DAG *D* on vertex set *X* in such a way that the sinks in *D* can be used as the backbone vertices of *T*. To make this work, it turns out that we first need to remove some vertices from *G* that have no effect on whether *G* is caterpillar-convex. First, we show that we can remove isolated vertices from *X* and vertices of degree 0 or 1 from *Y*.

### Lemma 2

Let $x\in X$ be a vertex with degree 0, and let $G'$ be the graph obtained from *G* by removing *x*. Then $G'$ is caterpillar-convex if and only if *G* is caterpillar-convex. Furthermore, a caterpillar representation of *G* can be constructed from a caterpillar representation of $G'$ by adding *x* in a suitable location.

### Proof

Let *T* be a caterpillar representation of *G*. If *x* is a leaf in *T*, we obtain a caterpillar representation $T'$ of $G'$ simply by removing *x* from *T*. If *x* is a backbone vertex in *T* with at least one leaf $\ell$ attached to it, we obtain $T'$ by replacing *x* in the backbone with $\ell$. If *x* is a backbone vertex in *T* without leaves attached to it, we obtain $T'$ by removing *x* and making the two former backbone neighbors of *x* adjacent (if *x* had two backbone neighbors).

For the other direction, let $T'$ be a caterpillar representation of $G'$. We can obtain a caterpillar representation *T* of *G* from $T'$ by adding *x* as a backbone vertex to one end of the backbone of $T'$. $\square$

### Lemma 3

Let $y\in Y$ be a vertex with degree 0 or 1, and let $G'$ be the graph obtained from *G* by removing *y*. Then $G'$ is caterpillar-convex if and only if *G* is caterpillar-convex. Any caterpillar representation of $G'$ is also a caterpillar representation of *G*.

### Proof

It is clear that any caterpillar representation *T* of *G* is also a caterpillar representation of $G'$. For the other direction, let $T'$ be a caterpillar representation of $G'$. The neighborhood of *y* induces an empty subtree or a single-vertex subtree in $T'$, so $T'$ is also a caterpillar representation of *G*. $\square$

We call a pair of vertices $x_i$ and $x_j$ *twins* if $N_G(x_i)=N_G(x_j)$. The twin relation on *X* partitions *X* into equivalence classes, such that $x_1,x_2\in X$ are twins if and only if they are in the same class. We say that two twins $x,x'$ are *special twins* if $\{x,x'\}$ is an equivalence class of the twin relation on *X* and if there is $y\in Y$ with $N_G(y)=\{x,x'\}$. Now, we show that removing a twin from *X* (with some additional modification in the case of special twins) has no effect on whether the graph is caterpillar-convex or not.

### Lemma 4

Let $x,x'\in X$ be twins of non-zero degree, and let $G'=(X'\cup Y',E')$ be the graph obtained from *G* by deleting *x*. If $x,x'$ are special twins in *G*, then modify $G'$ by adding a new vertex $\bar{x}$ to $X'$, a new vertex $\bar{y}$ to $Y'$, and the edges $\{x',\bar{y}\}$ and $\{\bar{x},\bar{y}\}$ to $E'$. Then *G* is caterpillar-convex if and only if $G'$ is caterpillar-convex. Furthermore, a caterpillar representation of *G* can be constructed from a caterpillar representation of $G'$ by adding *x* in a suitable location (and removing $\bar{x}$ if it has been added to $G'$).

### Proof

First, consider the case that *x* and $x'$ are not special twins. Assume that *G* is caterpillar-convex, and let *T* be a caterpillar representation. If at least one of *x* and $x'$ is a leaf in *T*, we can assume without loss of generality that *x* is a leaf (because the graph obtained from *G* by deleting *x* and the graph obtained by deleting $x'$ are isomorphic, as *x* and $x'$ are twins). In that case, removing *x* from *T* yields a caterpillar $T'$ that is a caterpillar representation of $G'$. Now assume that both *x* and $x'$ are backbone vertices in *T*. Form a caterpillar $T'$ by attaching the leaves in *L*(*x*) (where *L*(*x*) denotes the set of leaves attached to backbone vertex *x* in caterpillar *T*) as leaves to $x'$, removing *x*, and adding an edge between the two previous backbone neighbors of *x* (unless *x* was an end vertex of the backbone path). It is easy to see that $T'$ is a caterpillar representation of $G'$. Hence, in both cases it follows that $G'$ is caterpillar-convex.

For the other direction, assume that $G'$ is caterpillar-convex, with caterpillar representation $T'$. If $x'$ is a backbone vertex in $T'$, we attach *x* as leaf vertex to $x'$ in $T'$ to obtain a caterpillar representation *T* of *G*. If $x'$ is a leaf in $T'$ but $G'$ contains a twin $x''$ of $x'$ that is a backbone vertex in $T'$, we attach *x* as leaf vertex to $x''$ in $T'$ to obtain a caterpillar representation *T* of *G*. It remains to handle the case that $x'$ and all its twins (if any) in $G'$ are leaf vertices in $T'$. If $x'$ has a twin $x''$ in $G'$, this means that there is no $y\in Y'$ with $N_{G'}(y)=C$, where *C* is the equivalence class of $x'$ in $X'$; otherwise, $N_{G'}(y)$ would not be connected in $T'$. Therefore, there is also no $y\in Y$ with $N_G(y)=C\cup \{x\}$. Thus, if we denote by *b* the backbone vertex in $T'$ to which $x'$ is attached, we must have $b\in N_{G'}(y)$ for every $y\in Y'$ with $C \subseteq N_{G'}(y)$, and there must be at least one such *y* as $x'$ has non-zero degree. This implies $b\in N_G(y)$ for every $y\in Y$ with $C\cup \{x\}\subseteq N_G(y)$. Then we can attach *x* as leaf vertex to that backbone vertex *b* in $T'$ to obtain a caterpillar representation *T* of *G*. If $x'$ does not have a twin in $G'$, we know that $\{x,x'\}$ is an equivalence class in *X* and there is no $y\in Y$ with $N_G(y)=\{x,x'\}$ (otherwise, *x* and $x'$ would be special twins). Thus, if we denote by *b* the backbone vertex in $T'$ to which $x'$ is attached, we must have $b\in N_{G'}(y)$ for every $y\in Y'$ with $x'\in N_{G'}(y)$. This implies $b\in N_G(y)$ for every $y\in Y$ with $\{x,x'\}\subseteq N_G(y)$. Then we can attach *x* as leaf vertex to that backbone vertex *b* in $T'$ to obtain a caterpillar representation *T* of *G*. Hence, it follows that *G* is caterpillar-convex.

Now, we deal with the case that *x* and $x'$ are special twins. First, assume that *G* is caterpillar-convex, with caterpillar representation *T*. As there is $y\in Y$ with $N_G(y)=\{x,x'\}$, it is not possible that both *x* and $x'$ are leaves in *T*. Without loss of generality, assume that $x'$ is a backbone vertex. To obtain $T'$ from *T*, proceed as follows. First, if *x* is a leaf, remove *x* from *T*, and if *x* is a backbone vertex, attach all the leaves in *L*(*x*) to $x'$, remove *x*, and make the previous backbone neighbors of *x* adjacent to each other (only in case *x* was not an end vertex of the backbone). Then, add $\bar{x}$ as a leaf attached to $x'$. Observe that $N_{G'}(\bar{y})=\{x',\bar{x}\}$ induces a connected subgraph of $T'$. Therefore, $T'$ is a caterpillar representation of $G'$, and so $G'$ is caterpillar-convex.

For the other direction, assume that $G'$ is caterpillar-convex, with caterpillar representation $T'$. If $x'$ is a leaf vertex in $T'$, then it must be attached to the backbone vertex $\bar{x}$ (as $N_{G'}(\bar{y})=\{x',\bar{x}\}$). Furthermore, the only vertex in $Y'$ that is adjacent to $\bar{x}$ is $\bar{y}$. Therefore, we can swap the positions of $x'$ and $\bar{x}$ in $T'$, and the resulting tree is still a caterpillar representation of $G'$. Hence, we can assume that $x'$ is a backbone vertex in $T'$. To obtain a caterpillar representation *T* of *G*, we add *x* as a leaf attached to $x'$ to $T'$, and we remove $\bar{x}$: If $\bar{x}$ is a leaf vertex, we simply remove it, and if $\bar{x}$ is a backbone vertex, we attach all the leaves in $L(\bar{x})$ to an arbitrary other backbone vertex (for example, to $x'$), remove $\bar{x}$, and make the two previous backbone neighbors of $\bar{x}$ adjacent to each other (unless $\bar{x}$ was an end vertex of the backbone). The latter operation is correct as the only vertex in $Y'$ that is adjacent to $\bar{x}$ is $\bar{y}$. The resulting tree is a caterpillar representation of *G*. $\square$

We remark that the special treatment of special twins in Lemma 4 is necessary because there is a graph $G=(X\cup Y,E)$ with special twins that does not have a caterpillar representation $T=(X,F)$, while simply removing one of the two special twins (without adding the extra vertices $\bar{x}$ and $\bar{y}$) would produce a graph $G'=(X'\cup Y',E')$ that has a caterpillar representation $T'=(X',F')$. An example of such a graph is the graph with $X=\{a,b,c,f,g,x,x'\}$ where the neighborhoods of the vertices in *Y* are $\{a,f\}, \{a,b\}, \{b,x,x'\}, \{x,x'\}, \{b,c\}, \{c,g\}$. Here, the vertices *x* and $x'$ are special twins, and the graph obtained after removing *x* has the caterpillar representation with backbone path *abc* and leaf *f* attached to *a*, leaf $x'$ attached to *b*, and leaf *g* attached to *c*.

Let $G_1=(X_1\cup Y_1,E_1)$ be the graph obtained from $G=(X\cup Y,E)$ by removing vertices of degree 0 from *X*, vertices of degree 0 or 1 from *Y*, and twins from *X* (with the extra modification detailed in Lemma 4 in case of special twins) as long as such vertices exist. Lemmas 2–4 imply:

### Corollary 2

$G_1$ is caterpillar-convex if and only if *G* is caterpillar-convex.

We now define a directed graph $D=(X_1,A)$ based on $G_1$: For every pair of distinct vertices $x,x'\in X_1$, we let *D* contain the arc $(x,x')$ if and only if $N_{G_1}(x)\subseteq N_{G_1}(x')$, i.e., we add the arc $(x,x')$ if and only if every vertex in *y* that is adjacent to *x* in $G_1$ is also adjacent to $x'$ in $G_1$. Note that *D* is transitive: If it contains two arcs $(x,x')$ and $(x',x'')$, it must also contain $(x,x'')$.

### Lemma 5

*D* is a directed acyclic graph.

### Proof

Assume there is a cycle on vertices $x_i, x_{i+1}, \ldots , x_j$ in *D*. Then, $N(x_i)\subseteq N(x_{i+1}) \subseteq \cdots \subseteq N(x_j)\subseteq N(x_i)$. Thus $N(x_i)=N(x_{i+1})= \cdots = N(x_j)$, and so $x_i, x_{i+1}, \ldots , x_j$ are twins, a contradiction because there are no twins in $X_1$. Thus *D* is acyclic. $\square$

### Lemma 6

If $G_1=(X_1\cup Y_1, E_1)$ is caterpillar-convex, there is a caterpillar representation $T_1=(X_1, F)$ in which no two backbone vertices are connected by an arc in *D*.

### Proof

Let $T_1=(X_1, F)$ be a caterpillar representation for $G_1$ in which there are two backbone vertices $x_i$ and $x_j$ that are connected by an arc $(x_i,x_j)$ in *D*. If $x_i$ and $x_j$ are not adjacent on the backbone path $P_1$ of $T_1$, observe that $x_i$ also has an arc to every vertex between $x_i$ and $x_j$ on $P_1$. This is because, in $G_1$, each neighbor of $x_i$ is also adjacent to $x_j$ and hence to all vertices between $x_i$ and $x_j$ on $P_1$. Thus, we can choose $x_i$ and $x_j$ to be adjacent backbone vertices that have an arc $(x_i,x_j)$ in *D*.

**Fig. 4:**
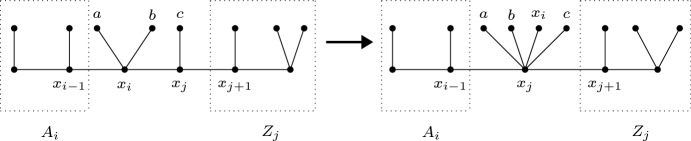
Caterpillar transformation from $T_1$ (left) to $T_1'$ (right) used in the proof of Lemma 6

Let *L*(*b*) denote the set of leaf vertices attached to a backbone vertex *b* in $T_1$. Observe that each leaf $\ell \in L(b)$ must have an arc to *b* in *D*. This is because each neighbor (in $G_1$) of $\ell$ has degree at least 2 and is hence also adjacent to *b*. Therefore, every leaf in $L(x_i)$ has an arc to $x_i$ and, by transitivity of *D*, also an arc to $x_j$. We now create a new caterpillar $T_1'$ from $T_1$ by (1) attaching $x_i$ and all the leaves in $L(x_i)$ as leaves to $x_j$, and (2) making the two previous backbone neighbors of $x_i$ adjacent (only if $x_i$ was not an end vertex of the backbone path). See Fig. 4 for an illustration.

We will show in the remainder of this proof that $T_1'$ is a caterpillar representation of $G_1$. By applying the same operation repeatedly as long as there exist two backbone vertices that are connected by an arc in *D*, the statement of the lemma follows.

Assume the vertices on the backbone path of $T_1$ are $x_1,x_2,\ldots ,x_i,x_j,\ldots ,x_r$ with $j=i+1$. Define $A_i=\bigcup _{k=1}^{i-1}(\{x_k\}\cup L(x_k))$ and $Z_j=\bigcup _{k=j+1}^r(\{x_k\}\cup L(k_k))$. These are the parts of the backbone that are not affected by the transformation from $T_1$ to $T_1'$. Note that $A_i$ and/or $Z_j$ can also be empty. We now prove for every $y\in Y$ that *N*(*y*) induces a tree in $T_1'$:

**Case 1:** $N(y) \cap (\{x_i, x_j\} \cup L(x_i) \cup L(x_j)) = \emptyset$. Since *N*(*y*) induces a tree in $T_1$, we must have $N(y)\subseteq A_i$ or $N(y)\subseteq Z_j$, and hence *N*(*y*) also induces a tree in $T_1'$.

**Case 2:** $N(y) \cap (\{x_i, x_j\} \cup L(x_i) \cup L(x_j)) \ne \emptyset$. Note that $x_j\in N(y)$ as all the vertices in $\{x_i\}\cup L(x_i)\cup L(x_j)$ have an arc to $x_j$ in *D*. As *N*(*y*) induces a tree in $T_1$, we observe that $N(y) \cap A_i$ is either empty or contains $x_{i-1}$, and that $N(y) \cap Z_j$ is either empty or contains $x_{j+1}$. As the caterpillar part on $A_i$ and $Z_i$ has not changed in the transformation from $T_1$ to $T_1'$, $N(y)\cap A_i$ is either empty or induces a tree containing $x_{i-1}$ in $T_1'$, and $N(y)\cap Z_j$ is either empty or induces a tree containing $x_{j+1}$ in $T_1'$. Furthermore, $N(y)\setminus (A_i\cup Z_j)$ contains $x_j$ and some subset of the leaf neighbors of $x_j$ in $T_1'$ and hence induces a star containing $x_j$ in $T_1'$. As $x_j$ is adjacent to $x_{i-1}$ and $x_{j+1}$ (if those vertices exist), *N*(*y*) induces a tree in $T_1'$. $\square$

### Lemma 7

If $G_1=(X_1\cup Y_1, E_1)$ is caterpillar-convex, there is a caterpillar representation $T_1=(X_1, F)$ such that the set of backbone vertices is exactly the set of sinks in *D*.

### Proof

By Lemma 6, there exists a caterpillar representation $T_1$ of $G_1$ in which no two backbone vertices are connected by an arc in *D*. Furthermore, every leaf attached to a backbone vertex (in $T_1$) has an arc (in *D*) to that backbone vertex (because every $y\in Y$ has degree at least 2). A backbone vertex cannot have an arc (in *D*) to a leaf attached to it (in $T_1$), as *D* is acyclic (Lemma 5). Finally, a backbone vertex *b* cannot have an arc (in *D*) to a leaf vertex $\ell$ attached to a different backbone vertex $b'$ because that would imply that *b* has an arc to $b'$ (since every vertex in *y* that is adjacent to *b* is also adjacent to $\ell$ and hence, as *N*(*y*) induces a tree in $T_1$, also to $b'$). Therefore, the backbone vertices of $T_1$ are exactly the sinks (vertices without outgoing edges) of *D*. $\square$

**Algorithm 2:**
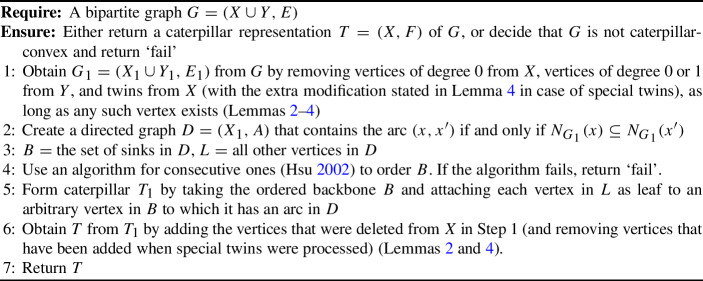
Recognition Algorithm for Caterpillar-Convex Bipartite Graphs

### Theorem 2

Algorithm 2 decides in polynomial time whether a given bipartite graph $G=(X\cup Y,E)$ is caterpillar-convex and, if so, outputs a caterpillar representation $T=(X,F)$.

### Proof

Let $G=(X \cup Y, E)$ be a bipartite graph. Let $n=|X\cup Y|$ and $m=|E|$. First, the algorithm removes vertices of degree 0 from *X*, vertices of degree 0 or 1 from *Y*, and twins from *X* (with the extra modification detailed in Lemma 4 in case of special twins) as long as such vertices exist. The resulting graph is $G_1=(X_1\cup Y_1,E_1)$. By Corollary 2, $G_1$ is caterpillar-convex if and only if *G* is caterpillar-convex.

We show that $G_1$ can be computed from *G* in $O(n^2)$ time. If *G* is given as adjacency matrix, we can compute an adjacency list representation in $O(n^2)$ time. Then, in $O(n+m)$ time, we can compute the degree of every vertex and make a list $L_0$ of vertices in *X* with degree 0 and vertices in *Y* of degree 0 or 1. As long as $L_0$ is non-empty, we remove a vertex *v* from $L_0$, decrease the degree of its neighbor (if any) by 1, and delete *v* from *G*. If *v* had a neighbor (which is only possible if $v\in Y$) and that neighbor now has degree 0, we add that neighbor (which must be in *X*) to $L_0$. This takes *O*(*n*) time as each vertex removed from $L_0$ can be processed in *O*(1) time and we remove at most *n* vertices. Let $X'$ and $Y'$ denote the vertices that have not yet been deleted at this stage. Next, we compute a partition of $X'$ into equivalence classes, where *x* and $x'$ are in the same equivalence class if and only if they are twins in the current graph. This can be done in $O(n^2)$ time. For example, one can start with a partition $\mathcal {P}$ consisting of a single equivalence class equal to $X'$ and then, for each $y\in Y'$, refine $\mathcal {P}$ in *O*(*n*) time so that each equivalence class *C* with $N(y)\cap C\ne \emptyset$ and $C\setminus N(y)\ne \emptyset$ gets split into $N(y)\cap C$ and $C\setminus N(y)$. During that process, we can also determine for each resulting equivalence class *C* whether there is a vertex $y\in Y'$ with $N(y)=C$, without exceeding the time bound of $O(n^2)$. Then, for each equivalence class *C* of size at least 2, we remove all but one vertex in *C* from $X'$ and update the degrees of the neighbors of the deleted vertices accordingly; the vertices in $Y'$ whose degree becomes 1 in this process are again added to the list $L_0$ and then deleted from the graph in the same way as above. Besides, if $x'$ is the vertex in *C* that we do not remove and if there is a $y\in Y'$ with $N(y)=C$, we add new vertices $\bar{x},\bar{y}$ and edges $\{\bar{x},\bar{y}\}$ and $\{x',\bar{y}\}$ to the graph in order to implement the treatment of special twins according to Lemma 4. We note that processing one equivalence class *C* in this way does not alter the other equivalence classes. In particular, it cannot happen that two other equivalence classes $C_1$ and $C_2$ get merged because the processing of *C* leads to the deletion of some vertices with degree 1 in $Y'$: when processing *C*, the vertices deleted from $Y'$ are not adjacent to any vertex outside *C*, and hence their deletion has no effect on the twin relationship between vertices outside *C*. The time for processing one equivalence class *C* can be bounded by the sum of the degrees of the vertices in *C*, and hence the time for processing all equivalence classes is bounded by $O(m)\subseteq O(n^2)$. Thus, $G_1$ can be obtained in $O(n^2)$ time. Note that $G_1$ has *O*(*n*) vertices as the number of new vertices added to $G_1$ is bounded by 2*s*, where $s\le \frac{n}{2}$ is the number of equivalence classes *C* with $|C|\ge 2$ for which a vertex $y\in Y'$ with $N(y)=C$ exists.

Next, the algorithm constructs the directed graph $D=(X_1,A)$ from $G_1=(X_1\cup Y_1, E_1)$ by adding an arc $(x_i,x_j)$ for $x_i,x_j\in X_1$ if $N_{G_1}(x_i)\subseteq N_{G_1}(x_j)$. For any two vertices $x_i,x_j\in X_1$ one can trivially check in *O*(*n*) time whether $N(x_i)\subseteq N(x_j)$, so *D* can easily be constructed in $O(n^3)$ time. As $|X_1|=O(n)$ and $|A|=O(n^2)$, the set *B* of sinks and the set *L* of remaining vertices can be determined in $O(n^2)$ time once *D* has been constructed.

Once the set *B* has been determined, we create a set system $\mathcal {S}$ containing for every $y\in Y$ the set $N(y)\cap B$ and apply an algorithm for checking the consecutive ones property (Hsu 2002) to check if *B* can be ordered in such a way that every set in $\mathcal {S}$ consists of consecutive vertices. If so, the resulting order is used to determine the order in which *B* forms the backbone path. Otherwise, $G_1$ (and hence *G*) cannot be caterpillar-convex (cf. Lemma 7), and the algorithm returns ‘fail’. As the input to the consecutive ones algorithm can be represented as a matrix of size $O(n^2)$, running the linear-time algorithm by Hsu (2002) on $\mathcal {S}$ takes $O(n^2)$ time.

Next, the algorithm attaches each vertex $\ell \in L$ as a leaf to an arbitrary vertex $b\in B$ to which it has an arc in *D*. It can be shown as follows that every $\ell \in L$ must indeed have at least one arc to a vertex in *B*: As *D* is acyclic, every vertex $\ell$ that is not a sink must have a directed path leading to some sink *b*, and as *D* is transitive, the arc $(\ell ,b)$ must exist. Attaching $\ell$ to *b* yields a valid caterpillar representation for the following reason: As every neighbor *y* of $\ell$ is also adjacent to *b*, and as $N(y)\cap B$ is a contiguous segment of *B*, it is clear that *N*(*y*) induces a tree in the resulting caterpillar $T_1$. Hence, $T_1$ is a caterpillar representation of $G_1$. Attaching the vertices of *L* as leaves to suitable vertices in *B* can easily be done in $O(n^2)$ time.

Finally, the vertices that have been deleted in the first step are added back (and vertices that have been added when special twins were processed are removed) in order to extend the caterpillar $T_1$ to a caterpillar representation *T* of *G*. This can easily be done in *O*(*n*) time per vertex following the arguments used in the proofs of Lemmas 2 and 4. By Corollary 2, *T* is a caterpillar representation of *G*.

The running-time of the algorithm is dominated by the time for constructing *D*, which we have bounded by $O(n^3)$. We remark that we have not attempted to optimize the running-time, as our main goal was to show that caterpillar-convex bipartite graphs can be recognized in polynomial time. $\square$

Finally, we discuss the case that the bipartition of the vertex set *V* of the input graph $G=(V,E)$ into independent sets *X* and *Y* is not provided as part of the input. First, if $G=(V,E)$ is a connected bipartite graph, note that there is a unique partition of *V* into two independent sets *Q* and *R*. We can then run the recognition algorithm twice, once with $X=Q$ and $Y=R$ and once with $X=R$ and $Y=Q$. *G* is caterpillar-convex if and only if at least one of the two runs of the algorithm produces a caterpillar representation. If $G=(V,E)$ is not connected, let $H_1,\ldots ,H_r$ for some $r>1$ be its connected components. As just discussed, we can check in polynomial time whether each connected component $H_j$, $1\le j\le r$, is a caterpillar-convex bipartite graph. If all *r* connected components are caterpillar-convex, the whole graph *G* is caterpillar-convex, and a caterpillar representation can be obtained by concatenating the backbones of the caterpillar representations of the connected components in arbitrary order. If at least one of the connected components, say, the component $H_j$, is not caterpillar-convex, then *G* is not caterpillar-convex either. This can be seen as follows: Assume for a contradiction that *G* is caterpillar-convex while $H_j$ is not caterpillar-convex. Then let $T=(X,F)$ be a caterpillar representation of *G*. Observe that the subgraph of *T* induced by $V(H_j)\cap X$, where $V(H_j)$ denotes the vertex set of $H_j$, must be connected. Therefore, that subgraph of *T* provides a caterpillar representation of $H_j$, a contradiction to our assumption. This establishes the following corollary.

### Corollary 3

There is a polynomial-time algorithm that decides whether a given bipartite graph $G=(V,E)$ is caterpillar-convex, i.e., whether it admits a bipartition of *V* into independent sets *X* and *Y* such that there is a caterpillar representation $T=(X,F)$.

## Conclusion

Determining the computational complexity of Li *k*-col for $k\ge 3$ when restricted to comb-convex bipartite graphs was stated as an open problem by Bonomo-Braberman et al. (2021). Subsequently, they proved that the problem is NP-complete for $k\ge 4$ (Bonomo-Braberman et al. 2024), but the complexity for $k=3$ was still left open. In this paper, we resolve this question by showing that Li 3-col is solvable in polynomial time even for caterpillar-convex bipartite graphs, a superclass of comb-convex bipartite graphs.

Recall that if mim-width is bounded and quickly computable for a graph class $\mathcal {G}$, then Li *k*-col is polynomially solvable when it is restricted to $\mathcal {G}$. Polynomial-time solvability of Li *k*-col on circular convex graphs is shown by demonstrating that mim-width is bounded and quickly computable for this graph class  (Bonomo-Braberman et al. 2021). On the other hand, there are graph classes for which Li 3-col is tractable but mim-width is unbounded, such as star-convex bipartite graphs (Bonomo-Braberman et al. 2024). By combining our result with Theorem 3 from Bonomo-Braberman et al. (2021), we conclude that caterpillar-convex bipartite graphs and comb-convex bipartite graphs also belong to this type of graph classes. On a much larger graph class, chordal bipartite graphs, the computational complexity of Li 3-col is still open (Huang et al. 2015).

Finally, as for future work, it would be interesting to see whether one can modify and extend Algorithm 2 to recognize comb-convex bipartite graphs.
